# Feasibility of conducting qualitative research with persons living with dementia and their caregivers during a home-delivered meals pilot trial

**DOI:** 10.1186/s40814-023-01302-5

**Published:** 2023-04-22

**Authors:** Emily A. Gadbois, Jennifer N. Bunker, Michelle Hilgeman, Renee Shield, Kathleen E. McAuliff, Whitney Mills, Kali Thomas

**Affiliations:** 1 Brown University School of Public Health, Providence, RI USA; 2 Research and Development Service, Tuscaloosa Veterans Affairs Medical Center, Tuscaloosa, AL USA; 3 Department of Psychology & Alabama Research Institute on Aging, The University of Alabama, Tuscaloosa, AL USA; 4 Providence VA Medical Center, Providence, RI USA

**Keywords:** Alzheimer’s disease and related dementias, Home-delivered meals, Caregivers, Pilot intervention, Recruitment, Informed consent, Cognitive assessment

## Abstract

**Background:**

Among older adults, food insecurity is associated with poor health status and health outcomes; people living with dementia (PLWD) are at increased risk for insecurity. Approaches to addressing food insecurity among homebound older adults include two modes of home-delivered meals: (1) meals delivered daily to participants’ homes by a volunteer or paid driver who socializes with the client or (2) frozen meals that are mailed to participants’ homes. Research has not examined benefits of these meals for PLWD or their caregivers nor compared the effectiveness of these two approaches in reducing food insecurity. The objective of this study was to test the processes for recruiting and engaging in qualitative research with PLWD and caregivers in an effort to understand the context, implementation, and mechanisms of impact by which relationships between meal delivery and outcomes may be achieved in preparation for a larger, follow-on study.

**Methods:**

This is a qualitative sub-study of a pilot, multisite, two-arm pragmatic feasibility trial comparing the effect of two modes of meal delivery on nursing home placement among 243 PLWD. In this sub-study, we tested recruitment and enrollment procedures and piloted interview guides among a subset of participants and caregivers.

**Results:**

We recruited and conducted interviews with nine PLWD and seven caregivers. In testing the informed consent process, all participants were able to consent to be interviewed, and PLWD all demonstrated capacity to consent. We successfully used a cognitive screener to obtain scores of cognitive impairment for PLWD and observed scores indicating a broad range of function. Our interview guides successfully resulted in information about the context, implementation, and mechanisms of impact for meal delivery during the pilot.

**Conclusions:**

In addition to establishing feasibility for the future trial, the substantive findings identified through the qualitative interviews provide an initial understanding of the contextual factors for meal delivery and the potential mechanisms of impact across meal delivery types that warrant further examination in a full-scale trial. Findings from our study provide crucial pilot data to support a follow-on trial to understand how to address food insecurity among PLWD.

**Name of the registry:**

ClinicalTrials.gov

**Trial registration:**

NCT04850781

**Date of registration:**

April 20, 2021, retrospectively registered

https://clinicaltrials.gov/ct2/show/NCT04850781

**Supplementary Information:**

The online version contains supplementary material available at 10.1186/s40814-023-01302-5.

## Key messages regarding feasibility

- Before we can test the differential impact of two standard approaches to home-delivered meals on outcomes for persons living with dementia (PLWD) in a large pragmatic trial, we must test the processes for recruiting and engaging in qualitative research with PLWD and their caregivers to understand elements that are not readily available in administrative data (i.e., context, implementation, mechanisms).
- We were able to successfully recruit PLWD and their caregivers for telephone interviews to understand the context, implementation, and mechanisms of impact for further examination in a larger trial.
- Taken together, these findings provide support for the feasibility of our methods and processes to be used in a follow-on trial. Explicitly, both opt-in and opt-out methods for recruiting PLWD and caregivers were successful and feasible, all participants were able to consent to be interviewed, all PLWD demonstrated capacity to consent, the cognitive assessment used determined that PLWD varied along a continuum of cognitive impairment, and our interview guide generated valuable information about individuals’ perspectives and experiences.

## Background

Among older adults, food insecurity is associated with poor health status and health outcomes and accounts for an estimated $130 billion annually in healthcare expenses [1]. People living with dementia (PLWD) are at increased risk of food insecurity [2–5]. A common approach to addressing food insecurity among older adults is providing home-delivered meals, which, in addition to reducing food insecurity, promotes socialization, health, and wellbeing [6–8]. Approximately 30% of home-delivered meals clients are PLWD [9]. While the benefits of home-delivered meals for older adults in general are well-understood, research has yet to explore the impact of receiving meals on PLWD.

Given the link between food insecurity and poor health, and associated increases in utilization and healthcare expenditures, health care entities are increasingly providing meals to their beneficiaries [10–16]. Home-delivered meals have traditionally been provided by volunteer or paid delivery drivers who bring one or more meals per day, often spending a few minutes chatting with the recipient and providing an informal wellness check. However, a lower-cost alternative has arisen, whereby multiple weeks of frozen meals, providing the same nutritional content, are delivered to recipients by mail. Unlike meals delivered daily that are ready for immediate consumption, frozen meals require additional steps for food storage and preparation that require cognitive abilities (e.g., prospective memory, cognitive sequencing) that are often impacted among PLWD. No research has yet compared the effectiveness of these two approaches in reducing food insecurity among older adults, or the experiences of recipients living with dementia.

We are planning to test the differential impact of these two standard approaches to meal delivery among PLWD in a large pragmatic trial. However, there are elements that we are unable to measure in with administrative data that require interviews with participants and caregivers. While these interviews make the study less pragmatic, they are needed to understand the context in which the interventions are delivered, implementation of the interventions, and mechanisms of impact and how those might differ between the two approaches. Therefore, the objective of this feasibility pilot is to test the processes for recruiting PLWD and their caregivers for interviews that we intend to scale up and implement in the follow-on, larger pragmatic trial.

## Methods

### Design

This is a qualitative sub-study of a pilot, multisite, two-arm pragmatic feasibility trial comparing the effect of two modes of meal delivery on nursing home placement among people with dementia (NCT# NCT04850781). The pilot trial enrolled 243 individuals on waiting lists at three Meals on Wheels programs in Florida and Texas to receive either (1) meals delivered multiple times per week by a Meals on Wheels volunteer or paid driver who may have socialized with the participant and provided an informal wellness check or (2) frozen meals that were mailed to participants’ homes every 2 weeks. The primary outcome of the pilot trial was the time to nursing home placement, ascertained using the nursing home Minimum Data Set. After 6 months, participants received their preferred meal from the participating programs [17].

In this qualitative sub-study, we tested the recruitment and enrollment procedures, as well as piloted the interview guides among a subset of participants and caregivers. In addition to informing the procedures for a future larger pragmatic trial, this qualitative sub-study provides additional insights into the experiences of receiving meals among a subset of participants living with dementia and caregivers.

### Participants

We sought to recruit a random subset of 6–12 participants (2–4 participants at each program) to participate in a telephone interview approximately 1 month after they began receiving meals. Inclusion criteria for the pilot trial included (1) being on a Meals on Wheels waiting list at one of three Meals on Wheels programs, (2) age 66 years or older, (3) a self-reported diagnosis of memory loss, cognitive impairment, or Alzheimer’s disease or related dementias, and (4) residence in an area where they could receive daily home-delivered meals. Participants were recruited into the qualitative sub-study from June to November 2021 if they were English-speaking and able to give consent to participate in the interviews (described below).

In addition to participants living with dementia, we aimed to recruit 6–12 caregivers of participants to take part in a separate telephone interview. Caregiver inclusion criteria for the qualitative sub-study included the following: (1) identification by the study participant as being a caregiver and (2) English-speaking.

### Procedures

#### Interviewer training

The interview team comprised of female, doctorate-level qualitative researchers (EAG, KEM, and RRS) with extensive combined qualitative interview experience of vulnerable populations, older adults, and caregivers. At the time of the interviews, EAG, KEM, and RRS were employed as assistant professor, project coordinator, and professor, respectively. Prior to recruiting participants, members of the research team (KEM, EAG, and RRS) participated in a specialized training focused on conducting interviews with PLWD. Led by a licensed clinical geriatric psychologist, this two-part training included a review of informed consent procedures, capacity to consent, administering and scoring the Modified Telephone Interview for Cognitive Status (TICS-M) [18, 19], and the logistics of conducting a phone interview with PLWD. Additionally, this training covered the symptoms of dementia and how these symptoms may impact qualitative interviews. Best practices on how to effectively communicate with PLWD, including strategies to preserve dignity and reduce frustration during parts of the interview sometimes considered challenging for the participant, were also emphasized.

#### Recruitment for qualitative sub-study

##### Participants living with dementia

We set recruitment goals for a random subset of 6–12 participants (2–4 participants at each program) to take part in a telephone interview approximately 1 month after they began receiving meals. Interview participants received a $50 gift card to the pharmacy of their choice for participating. In order to determine which recruitment approach would work best for the larger study, we tested two different methods: opt-in and opt-out. For both methods, we had data use agreements with the programs, which provided the research team with contact information for eligible clients.

Opt-in We tested an opt-in method for recruiting participants at two sites: program 1 [Neighborly Care Network] and program 2 [Visiting Nurse Association of Texas]. The research team mailed a welcome letter, an informed consent sheet, and contact information for the research team to the participant’s address on file. Interested participants were able to contact the research team directly to schedule an interview or to learn more about the qualitative sub-study.

Opt-out We tested an opt-out approach for recruiting participants at a third site: program 3 [Meals on Wheels of San Antonio]. The research team mailed each participant a welcome letter, an informed consent sheet and contact information for the research team. In contrast with the opt-in method, the opt-out letter offered an opportunity for potential participants to either contact the research team directly by phone or email to opt-out of being contacted or to return a pre-addressed, pre-stamped “opt-out” postcard. Participants had a minimum of 2 weeks after the letter’s postmarked date to opt out before they were contacted by the research team. Additionally, we informed participants that if they did not opt out of being contacted, we would contact them no more than three times.

##### Caregivers

To identify potential caregivers, we asked participants living with dementia in the qualitative sub-study if they had someone who helped them out day-to-day, (for example, a spouse, child or someone else who helped them regularly) who might want to talk to us. We asked participants to share contact information of that caregiver who may be interested in being interviewed. The research team then called caregivers to see if they were interested in participating in a separate caregiver interview. Caregiver participants received a $50 gift card to the pharmacy of their choice for participating.

#### Informed consent

##### Participants living with dementia

We received a waiver of documentation of informed consent for the qualitative sub-study. A trained research team member reviewed the informed consent sheet and obtained verbal consent from the participant prior to the interview. Because we were enrolling participants living with dementia in the qualitative sub-study, we used an IRB-approved capacity to consent checklist to determine if the following criteria were met: (1) Did the individual make a “clear choice” to participate? (2) Did the individual show “understanding” of what they were consenting to do? (3) Did the individual describe “reasoning/rational reasons” for wanting to participate in the study? (4) Did they understand that participating is optional and that they can change their mind? If it was determined that the participant could describe in their own words the purpose, procedures, risks/benefits, and voluntary nature of the study, the interviewer scheduled an interview time with the participant that worked best for the participant’s schedule.

##### Caregivers

We mailed caregivers an informed consent sheet and obtained verbal consent prior to the interview. We did not formally assess caregivers’ capacities to consent to the research study as it was not expected that they may be unable to understand the purpose, procedures, risks/benefits, and voluntary nature of the study.

#### Interview procedures

A member of the research team called the participant 1 day prior to the interview as a reminder, confirmed their interest in participating, and also asked the participant if it was acceptable to record and have a note taker join the interview. Declining a note taker’s presence or audio recording did not affect eligibility to participate in the interview. During this reminder call, we also communicated to participants that we were interested in hearing from just the person being interviewed, for example, just the person living with dementia and not the caregiver. Interviews occurred by phone. No one else was present during interviews except for the participants and researchers (EAG, KEM, RRS). While we cannot be sure who may have been present in the background during interviews, at the start of each interview, we reminded participants that we were interested in hearing from them directly about their own experiences. Interviewers introduced themselves to participants at the start of each interview, reiterated the goals of the study, and the reasons for doing the research.

**Fig. 1:**
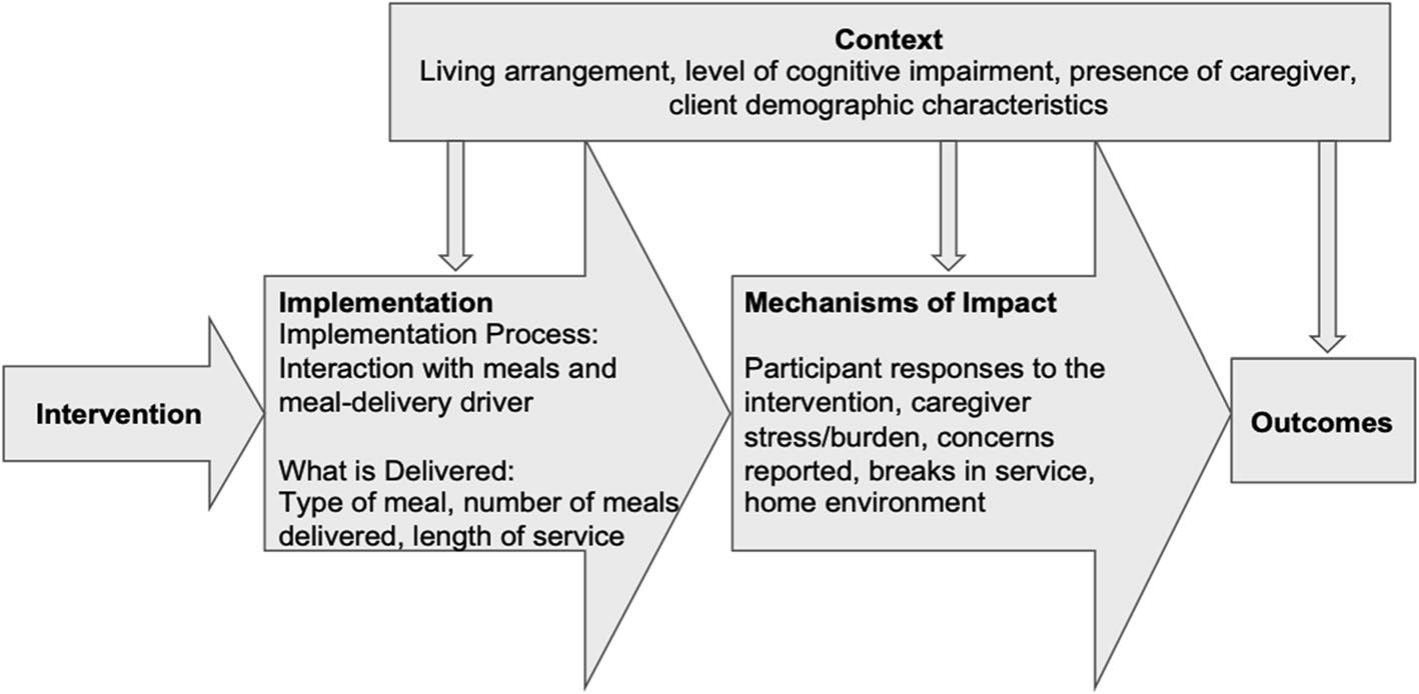
Adaptation of the Medical Research Council’s Process Evaluation Framework

The interview guides were developed using the Medical Research Council’s Process Evaluation Framework (see Fig. 1) [20]. In addition to gaining substantive content about participants’ and caregivers’ experiences receiving meals, we tested the feasibility of using these interviews to gather content based on the Process Evaluation Framework. This included “context” content that might influence how the meal intervention was received. We included questions about client participants’ living arrangements, level of cognitive impairment, presence of a caregiver, and demographics. “Implementation” content included questions about the timing and logistics of meal delivery and meal preparation, the number of meals received, and interactions with meal delivery drivers. “Mechanisms of impact” content included questions about participants’/caregivers’ satisfaction with the meals and their delivery, strengths and challenges of the intervention, and their overall experiences.

The semi-structured interview guides were developed to last approximately 30 min. While both daily and frozen participant interview guides focused on the participants’ experiences receiving meals and contained the same questions, the interview guide for frozen meal participants and the interview guide for daily meal participants used slightly different prompts for the questions about meal delivery logistics (see Additional File 1 for daily meal participants, Additional File 2 for frozen meal participants). The interview guide used for caregivers also included questions focused both on the participants’ experiences receiving home-delivered meals, and their experiences as a caregiver (see Additional File 3).

#### Modified Telephone Interview for Cognitive Status (TICS-M)

At the completion of the interview with participants living with dementia, the interviewer administered the Modified Telephone Interview for Cognitive Status (TICS-M). The TICS-M is a validated instrument, designed to be administered by phone. While the TICS-M is a useful screening tool for identifying cognitive impairment, it is not intended to replace a full cognitive battery of assessments, which would more precisely indicate an individual’s level of cognitive functioning. Total scores can range from 1 to 39, with a score of 21 or lower indicating mild cognitive dysfunction [21–23]. We used this instrument both to characterize participants’ cognitive functioning and to ensure that our subsample reflected adequate variability in dementia severity since we wanted to recruit and interview participants of varying levels of cognitive functioning.

#### Interviewer debrief and interview transcription

After each interview, the interviewer and note taker debriefed about the interview. These initial impressions and thoughts about the interview were compared and discussed, then captured in an interview audit trail [24]. Interview audio was transcribed by an independent third party. Repeat interviews did not occur and transcripts were not returned to participants for comment or correction. Participants did not provide feedback on the findings.

### Analysis

#### Interview analysis

After the interviews were completed, the qualitative research team met twice to discuss steps for data analysis. During the first meeting, we discussed our initial impressions from both the caregiver and participant interviews, using interview notes and the audit trail. During the second meeting, we discussed a strategy to analyze the data. We first drafted a coding scheme and coding definitions based on topics covered in the interview guide and informed by the content discussed during the interviews and reflected in the notes and the audit trail. The coding scheme is a heuristic mechanism to sort narrative text into discrete pieces so they can be compared and grouped. Then, each team member individually applied the coding scheme to one participant and one caregiver interview to test the feasibility of the structure. Team members reviewed the coded interviews and made revisions to the scheme. We re-coded the same two transcripts to test the revised scheme. Final edits were then made. The final scheme included coding categories of background/content, meal delivery, satisfaction with meals, before Meals on Wheels, other meal times, benefits of meals, challenges and changes, TICS-M, caregiver, and good quotes. The remaining 14 transcripts were double-coded by pairs such that each of the five coding team members coded between four and six transcripts. Coding of transcripts was managed in NVivo version R1.6. As interviews were coded, we continued to add discussion notes to the audit trail, including emerging ideas about patterns of responses. We assessed rigor through constant comparison with members of the qualitative research team; recording discussion and comparison notes with an audit trail helped clarify questions about the coding scheme, definitions, and interpretations. Throughout the analysis process, we met with the participating Meals on Wheels programs, the larger research team, and our study’s external Stakeholder Advisory Panel (comprised of meal delivery drivers, caregivers of PLWD, and PLWD) to share general impressions and interpretations of the findings and receive feedback and ideas. In addition to coding the substantive material of the interviews in this manner, we also assessed how well the interviews were able to generate data related to the Process Evaluation Framework, including in the domains of context, implementation, and mechanisms of impact. Because the primary goal was to determine feasibility of the qualitative sub-study, data saturation was not an objective of the analytic plan.

#### Feasibility analysis

We examined data to determine feasibility and inform the larger, follow-on trial. Table 1 below lists and explains our feasibility objectives, criteria for determining success, and associated data sources.

**Table 1:** Feasibility objectives, criteria for success, and data sources

	**Feasibility objectives**	**Criteria for success of feasibility**	**Data sources**
**PLWD recruitment**	To determine which of our two recruitment methods are the most successful in recruiting PLWD for interviews	Number of participants expressing interest in the interview Number of participants who completed the interview	Call log; recruitment log; call notes (dates, what was said, etc.), contact attempts; contact methods; meeting notes with research team and community partners
**Caregiver recruitment**	To determine to what extent our method of recruiting caregivers through PLWD was successful	Number of caregivers identified, contact information provided Number of caregivers who completed the interview	Call log; recruitment log; call notes (dates, what was said, etc.); contact attempts, contact methods; meeting notes with team and community partners
**Informed consent**	Test processes for obtaining informed consent from participants and caregivers	Number of participants who were able to complete the consent process	Informed Consent Checklist and corresponding notes; notes and Zoom recordings from meetings with team members and clinical psychologist; interview summary; audit trail
**TICS-M**	To determine the varying levels of cognition among participants To examine whether individuals with more significant impairment would be able to reflect on/provide feedback about the meal delivery process in advance of the larger trial	TICS-M scores Substantive responses regarding preferences/experiences across range of TICS-M scores	TICS-M scores; interview transcripts; interview summaries; audit trail
**Interview guide**	To determine if the items, including probes in the interview guide, elicit meaningful and appropriate responses to each of the items to help answer questions about the two methods of meal delivery	Able to gather content in the domains of context, implementation, and mechanisms of impact Questions are understood by interview participants, generate substantive responses	Interview transcripts; interview summaries; audit trail

## Results

### Recruitment

#### Participants living with dementia

A primary feasibility criterion was testing our two recruitment methods among participants. At the two programs where we used an opt-in approach, we mailed 56 recruitment packets. We received phone calls from six participants (program 1 *n* = 3, program 2 *n* = 3) who were interested in participating in the interviews. All six individuals completed an interview.

At the third site where we tested the opt-out approach, we mailed 42 recruitment packets to participants. We received responses on behalf of 10/42 eligible participants. Of the eligible participants, seven opted out, one client participant opted in and completed an interview, and two other client participants expressed interest but did not complete an interview; additionally, although we did not anticipate recruiting caregivers using these opt-in and opt-out methods, this initial opt-out approach yielded two completed caregiver interviews and one caregiver who expressed interest but did not complete an interview. We then contacted the 32 individuals who did not opt out or in during the prior step. Of those 32, nine client participants opted out, 13 were unreachable by telephone after repeated attempts, two completed an interview, three expressed interest but did not complete an interview, and five were not called as we had reached our goal sample. These outreach attempts also yielded five completed interviews with caregivers and one caregiver who expressed interest but did not complete an interview.

Thus, our sample of client interview participants included six participants recruited using the opt-in approach and three recruited using the opt-out approach. Additionally, although we did not plan for it, our opt-out approach yielded seven completed caregiver interviews.

#### Caregivers

In recruiting caregivers, our planned approach relied on participants living with dementia to identify caregivers and provide contact information to the research team. Four interview participants identified caregivers and provided caregiver contact information. However, those four caregivers were determined unreachable after three contact attempts. While we did not plan for another method of recruiting caregivers for interviews, as discussed above, we were able to recruit caregivers as a byproduct of our opt-out client participant recruitment, achieving our objective of completing between six and twelve interviews with caregivers. Our seven caregiver interviews included one husband, one wife, one daughter-in-law, three daughters, and one son of pilot participants.

### Informed consent

All 16 interview participants completed the consent process, and all nine PLWD were deemed to have the capacity to consent. The study team’s capacity to consent checklist effectively supported participants in understanding the study as well as the study team’s documentation of their capacity to consent to the interview.

### TICS-M

The TICS-M was successful for documenting a range of cognitive abilities over the phone for the nine PLWD. The scores in this sample ranged from 10 to 32. Participants scored an average of 22 out of 39 (SD = 7.14).

Our second feasibility objective with regard to the TICS-M was determining the extent to which individuals with more significant impairment would be able to provide feedback about their meal delivery experience. Four participants in our sample had scores of 21 or lower, indicating likely cognitive impairment [21, 25]. Despite their lower scores, these participants were able to successfully provide their perspectives and feedback about receiving meals. See Table 2 for example quotes from these participants.

**Table 2:** Example quotes from participants with cognitive impairment according to the TICS-M

**Participant ID (meal type)**	**TICS-M score**	**Example quote**
Participant 2 (daily)	10	“They mix the food. They don’t give me one thing all the time. Same thing over and over. They don’t give me, they mixed it, like how I would eat my food if I was cooking for myself.” “Sometimes I eat part of it and put away a part, but I don’t let it stay for the other day because I don’t like to let it stay for the other day, because I don’t know when they cook it. So, I like to have it at the same time when they get here with it.”
Participant 8 (frozen)	13	“I used to eat a lot with everything. I wasn’t thinking that I had to take care of myself, which is why I have diabetes. I try to be more careful. At the time, I had my husband, and he loved to eat, so I’d prepare everything for him. Homemade soup, lasagna. I would cook everything. On Christmas I would make tamales. And they’d always ask for more and more!” “I was in the hospital for a whole month and they didn’t want to let me go until I had someone to take care of me. I just have the one son, and he came. Dropped everything he was doing and came to live with me and help me out. I had a daughter, but she has a daughter of her own and they live out of town. So I didn’t have anyone. He’s taking care of me, medicines, groceries, things like that. He’s the oldest, and never been married. Too much trouble to get married [laughs]. Everyone says he’s smart, not getting married.”
Participant 6 (frozen)	20	“I ain’t got to worry about too much, because I eat one in the morning for breakfast, then eat me another one for lunch. If I don’t eat all of that, I can have leftovers for supper. But I eat pretty good. I can eat two a day real easy.” “[Before getting meals delivered] I kind of ran out a little bit, but since y’all helping me this way, I ain’t got to worry about too much. It was a little hard, but it’s a lot better now. It’s better now.”
Participant 7 (daily)	21	“They will never just leave the meal. And if I was to say, ‘Just leave it at the door and I'll pick it up.’ They don’t do that. I’ve never done that. They physically will hand off the meal.” “So, it’s very nice, because you can establish a rapport. On Tuesday, there’s a particular gentleman that comes, and so because there’s that one particular person that comes on Tuesday, you can establish a ready rapport. And so, it’s nice to have that familiar person, and so they know your routine.”

### Interview guides

Both participant and caregiver interview guides were able to elicit meaningful and appropriate responses to each of the items. The interview guides also successfully generated content based on the Process Evaluation Framework that allowed for understanding the context, implementation, and mechanisms of impact associated with the two types of meal delivery. Table 3 includes example quotes generated across domains of the framework.

**Table 3:** Example quotes and domains of the Process Evaluation Framework

**Domain**	**Example quote**
Context: Living arrangement	“Actually, she lives with me…The house that the meals are delivered to, it’s her house, but since she’s no longer allowed to stay by herself, she lives with me now.” (Caregiver 4; frozen) “I wish more people could get them because I know there’s a lot more people out there who need this service. I live in a retirement community and there’s probably five to 6,000 residents in this complex.” (Participant 1; frozen) “I stay here…since my dad passed away in January.” (Caregiver 5, daily)
Context: Level of cognitive impairment	“I can leave her for a little while. I’ll tell her. She won’t remember where I’ve gone. She has a very short memory because she has dementia. I go in and tell her before I leave, I’m leaving, and so she knows that I’m going be gone for a little while.” (Caregiver 2; daily) “I have a bad memory. That’s why the doctor giving me medicine for my brain.” (Participant 2; daily) “... She gave up driving, right at around 79 [years old]. And then she started showing signs of memory loss, cognitive abilities. You could tell things were starting to decline. And so that’s where I had to pay even more attention to her needs and making sure she was attending all her doctor appointments, scheduling those appointments, getting her there, following up, and then taking any kind of medication that was necessary.” (Caregiver 1; frozen)
Context: Presence of a caregiver	“I know my dad, he wouldn’t want her in a nursing home…Really it’s just me…I have friends that have offered me jobs and this and that, but I can’t leave her. To be here and then to have meals sent to us, to our door is just wonderful. That makes the possibility of her being able to be here a possibility.” (Caregiver 5; daily) “No, I don’t have anybody. I help myself alone here.” (Participant 2; daily) “It takes both of us to take care of Mom. And I’m really low income because of the disability amount I get- and how much we have to pay for rent here at the apartment. So, I have a caregiver that comes in and helps in the house, and it takes all three of us to take care of Mom.” (Caregiver 7; frozen)
Context: Demographics	“I do like them…I mean, they could be maybe, I like Mexican food because you know, I am Hispanic myself, but they’re fine. I appreciate them.” (Participant 9; frozen) “She has lived with us, her son being my husband and two daughters. It was time for her to retire and just be able to live without any issues, any worry so we invited her, just come and live with us. So she has lived with us for over, let’s see, 10 years now.” (Caregiver 1; frozen) “Right now, I’m VA disabled. And they don’t know that I’m taking my dad in because I’m afraid they’ll take my disability away. But at the same time, I told my husband if I decline because of dad being here, then I will make that decision that I don’t want to make.” (Caregiver 6; daily)
Implementation: Timing of meal delivery	“They usually come about 11:30.” (Caregiver 2; daily) “I just sit right here. They’ll tell me about what time they’ll be here. It be about the same time, around about 10:30 or 11:00. I really don’t know, but about the same time.” (Participant 6; frozen) “I didn’t get a call, and I thought, ‘Well, maybe they’re going to come at the same time.’ And they didn’t, so I just kept sitting up and waiting, and then I decided I’ve got to go take my nap, and it wasn’t until... I don’t know. The first time it was 2:30? And so, then after that, it was 1:00, and then after that, it’s been around 11:00, 11:30.” (Participant 7; daily)
Implementation: Logistics of meal delivery	“Well, someone, you know, comes to the door and, and rings the bell or knocks. And so then, you know, they have a bag with food in it, the meals and that’s it…They just stay at the door and hand it to you.” (Participant 4; daily) “If I knew when to expect it, for the most part I’m always going to be here... especially it’s getting hot and I can’t tell you, because it was always such a surprise to me, what time, but it’s a FedEx truck. FedEx that brings it. But if I knew ahead of time, that would be more helpful to me.” (Participant 5; frozen) “She just knows that they brought her something. We have a camera at her house... The last time…they brought the meals, she was over here with me. When we see them delivered, then we go pick them up.” (Caregiver 4; frozen)
Implementation: Meal preparation	“It makes it a little easier because I know that them meals would be here, and I won’t have to worry about whether the little home provider, if she could feed him because she already have those meals ready. I’m like, ‘All you have to do is warm it up and mash it up a little bit.’” (Caregiver 3; daily) “I just stick them in the freezer until I’m ready to eat them and follow the directions, slit over each compartment and nuke it for what they say. And I usually nuke it for probably about a minute longer because some of the vegetables sometimes don’t really get hot. So I just found out that if I just nuke it for an extra minute, it’s no problem.” (Participant 1; frozen) “They come in really handy and the only thing that you need to do... I don’t use the microwave because I don’t think it comes out quite as well. I use the other method, put it on low in the conventional, in the oven.” (Participant 5; frozen)
Implementation: Number of meals received	“They come on Monday, they give for three days, three meals and then they come on Wednesday and give me for two days.” (Caregiver 2; daily) “They come every other Friday…Every two weeks, and they’re enough for two weeks.” (Caregiver 4; frozen) “I think six meals came, I think it was extra meals because of maybe they’re taking off on Monday or maybe... I don’t know, I wonder. It seems like it was extra meals but I can’t quite figure out... I can’t remember.” (Caregiver 6; daily)
Implementation: Interactions with meal delivery drivers	“I don’t know how you guys find these happy people, but you guys have some happy people... One guy comes every day… He’s just always total happy and just joyful. It rubs off on you. When you see his smiling face, you want to smile, too. I’m like, ‘That’s really cool. They got a really good worker.’” (Caregiver 5; daily) “Sometimes we have a nice chat. Sometimes I said, ‘Hi.’ Or they say, ‘How are you doing? You are doing all right?’ I said, ‘Yes, I’m okay.’ They are nice people.” (Participant 2; daily) “They just bring them and knock on the door. And if I don’t answer, then they call me because it takes me a while to get out of bed. So I make it to the door…Well I’ve got a walker and they say, ‘Can I help?’ I said, ‘Just put it on the walker.’” (Participant 3; daily)
Mechanisms of impact: Satisfaction with meals/delivery	“The hardest part was what to fix. I don’t mind cooking it, but to come up with a menu, I guess, I should say. And the Meals on Wheels is well rounded. You get tired of it, but it’s still well rounded. You always get some kind of an entree, then you get vegetables, usually two vegetables. They also bring drinks, orange juice and apple juice. They seem to give you some for every day and some fruit. We’ve been getting bananas and apples. Which are very tasty and help.” (Caregiver 2; daily) “She only knows that the meals are delicious. She doesn’t know who cooked them. No, really, every time we feed her the meals, she’s like, ‘Oh, this is very tasty. You cooked this?’ She wants to know who cooks them. Yes. And she eats, I mean, the variety of the meals it’s excellent. It’s a lot of food and selections that she would normally eat. The chicken, the meatloaf, the fish with a lot of vegetables.” (Caregiver 1; frozen) “They’re good quality meals. I mean, it’s almost like going out to a restaurant... It’s not like a fast food thing. I mean, and they’re healthy, they’re nutritious, they’re low in calories, low in sodium. They’re very health conscious. I don’t find anything really wrong with them.” (Participant 1; frozen)
Mechanisms of impact: Benefits of meals	“I don’t have to buy all the food that it takes to make all the food that we get. Really, it’s a lot of money because you have a big variety....A lot of those vegetables or fruits or healthy stuff is expensive…If I had to go buy the food today, I just wouldn’t be able to, but she’s got three meals, so she’s good for another couple of days, too. Me, I’m fine with sandwiches. I’m okay, but my mom, she needs to have food that where it’s going to give her substance and nutrition and where she’ll have enough energy to get up and move around. If she doesn’t continue walking, then she will completely forget how to do it… I wouldn’t be able to afford all the vegetables and everything that you guys supply and bring.” (Caregiver 5; daily) “I would say it has affected, again, my stress level, worrying about, ‘Okay, I got to get her fed’ or, ‘Oh my god, what would I need to fix the day for her?’ Or what have you. So it has definitely helped to reduce a certain level of stress in my life, free up some time to spend with her otherwise." (Caregiver 1; frozen) “Because he has food and as long as I remind him to eat it, then he is able to maintain his strength, which keeps him from being weak, which keeps him from falling, which keeps him from being in the hospital. So food, it does provide safety in an indirect way.” (Caregiver 6; daily) “I think it has helped. Because before, I definitely don’t believe I was getting enough nutrition.” (Participant 7; daily)
Mechanisms of impact: Challenges with meals	“There’s a lot of stuff in there on the meals that are stuff Mom won’t eat. So, like beans and stuff, she doesn’t eat beans. And they’ll have stuffed manicotti, which she doesn’t like ricotta cheese. And fish, she will not tolerate, so she’s allergic to milk products if she eats too much, and we don’t do anything with oranges. She doesn’t like them... When it first started out, the hardest part was the fact that they were sending two boxes every other week, which meant I had a storage problem. Because that was 20 meals, trying to find a place to put them, and they finally got that straightened out. And what I’ve had to do the last couple of times is one of the small freezer boxes I got, I just put the refreezable packs in the freezer and just keep them in the box and keep the freezer packs in there because I just don’t have room in the freezer for that many meals. They just don’t fit.” (Caregiver 7; frozen) “If I’m not here, I put the bag out there. But when I put the bag out there and I’m not here, they don’t put the food in there. And if I leave a note, ‘I'll be right back. Please put the food.’ When I get here, the empty bag was there and I saw a note on it that they’re not supposed to leave the food if I’m not here.” (Participant 2; daily) “The only thing that was a bit challenging or stressful was the whole holiday thing. On that Monday. It’s like dad didn’t get the Monday delivery…So he went without meals and I didn’t know. And so then, I found out that if it’s a holiday on Monday, they don’t deliver. So I thought, ‘Okay, I have to plan ahead. If a holiday’s coming, I need to buy some TV dinners to have in there, to kind of be there for just in case.’ And I did that.” (Caregiver 6; daily)
Mechanisms of impact: Overall experience	“I really appreciate that I’m getting them because otherwise, you know, I don’t know how often I’d be eating.” (Participant 9; frozen) “It’s providing the value nutrition that she needs. The proteins, the minerals, vitamins from the vegetables, which in turn, it’s keeping her healthy. She does take vitamin supplements because obviously she can’t eat all the requirements of minerals and vitamins that’s necessary, but it really provides the nutrition and hence the energy she needs to keep living. And it was a very needed thing and it really just keeps her going.” (Caregiver 1; frozen) “The meals that we get? Well, generally they’re, they’re very good. And I know I like them.” (Participant 4; daily)

## Discussion

Overall, we determined that our processes for recruiting PLWD and caregivers for qualitative telephone interviews, obtaining consent, conducting a cognitive screen, and administering the interviews were feasible. In addition, our interviews enabled us to understand the context, implementation, and mechanisms of impact for further examination in a larger trial. Both the opt-in and opt-out methods for recruiting PLWD for interviews were successful and generated completed interviews (*n* = 6 opt-in, *n* = 3 opt-out). While our planned approach to recruit caregivers successfully yielded caregiver contact information, this did not lead to completed interviews. However, we learned that during the opt-out process of recruiting PLWD we were able to recruit caregivers. In testing our informed consent process, all participants were able to consent to be interviewed, and the nine PLWD all demonstrated capacity to consent. We successfully used the TICS-M to obtain scores of cognitive impairment for PLWD and observed scores ranging from 10 to 32, indicating a broad range of function. We also found that the four participants who were likely cognitively impaired according to the TICS-M were able to provide valuable information about their perspectives and experiences. Lastly, our interview guide successfully resulted in information about the context, implementation, and mechanisms of impact for meal delivery during the pilot. Taken together, these findings provide support for the feasibility of our methods and processes to be used in a follow-on trial.

The interviews with PLWD and caregivers generated critical information about the benefits and challenges of the two modes of meal delivery. Numerous benefits were reported for both daily and frozen meal recipients. Such benefits included convenience and that the meals saved both time and money and freed caregivers up to spend additional time with PLWD. Interview participants also describe health and nutritional benefits and that meals added greater variety to what recipients were eating. Daily meal recipients also described the benefit of positive interactions with their delivery drivers. Interviews also yielded challenges, including that those receiving frozen meals had to read instructions and operate a microwave to prepare meals, which is of particular relevance for those with cognitive impairments. Other challenges included confusion about the timing of deliveries, the challenge of storing large quantities of frozen meals, and having to be present for the meal delivery for those that received them daily. However, PLWD and caregivers were, overall, very satisfied with the meals they received.

Despite the success and feasibility, our pilot was not without challenges. In recruiting participants via the opt-out method, we encountered evidence of possible self-selection bias that made it challenging to directly reach potential participants living with dementia—particularly those with lower cognitive abilities. For example, caregivers often answered the phone and preferred that they participate in the interview, rather than the person receiving meals. When asked why they did not want their loved one to participate, they expressed concern about the meal recipient’s cognitive impairment. Examples of this include: “My dad is 90 years old and has Alzheimer’s, he won’t be able to finish the interview.” and “As my mom’s caregiver, I don’t think she can really give you feedback, as she has dementia, but I can talk to you.” As we were not able to conduct interviews with these PLWD, we are unable to determine if they would have had capacity to consent or the ability to complete the interview and provide information on their perspectives. The fact that none of the PLWD were unable to consent to the interview is further evidence that only higher functioning individuals were self-selecting to participate. Additionally, we had a relatively low response rate to our recruitment efforts, which must be considered in planning and budgeting for a larger trial.

We also encountered challenges during administration of the TICS-M. One such challenge was the role of hearing impairment. Even after including TICS-M-directed prompts that we could not repeat instructions or words for later recall, participants frequently requested that we repeat instructions or words for recall. Some participants were non-native English speakers, and/or those for whom the TICS-M’s language-based assessments were more challenging. Relatedly, we encountered challenges with regard to education. In responding to TICS-M questions, some participants remarked on having limited literacy and/or education. In such cases, we are unable to determine if challenges or incorrect responses were the result of cognitive impairment or of hearing impairment, phone connectivity issues, cultural/language barriers, or educational or literacy barriers. Additionally, in order to limit burden on participants, we did not gather detailed demographic data, including educational attainment, which would be needed in order to include an educational adjustment for those who did not complete high school. Not unique to this study or the TICS-M, the challenges we observed are consistent with known barriers to estimating cognitive functioning virtually using brief cognitive screeners [26].

An additional limitation may be that participants could have responded in socially desirable ways, given that the study enabled them to receive services sooner than would have otherwise been available. The fact that our evaluation team was independent and not fully embedded in the Meals on Wheels programs likely mitigates this risk. There is also not a reason to expect that social desirability demands would vary by meal type, which was supported by the fact that we did hear negative feedback during the course of these interviews across both arms of randomization (frozen and daily delivered meals).

Despite these limitations, our findings have important implications for research focused on supporting PLWD and their caregivers in the community. PLWD, including those with cognitive impairment according to the TICS-M, shared their experiences receiving meals. Consistent with current practice recommendations for including PLWD in research relevant to their interests and needs [27], this feasibility study builds on a growing body of literature about effective strategies to engage PLWD in research [28–34]. Of note, while we were able to safely conduct this research with the support of our institutional regulatory body, particular attention must always be paid to best practices to safeguard the wellbeing of PLWD participating in research [31]. We found that our methods of recruitment were successful in recruiting PLWD and caregivers, our informed consent and capacity to consent processes were feasible, the TICS-M effectively found a range of cognitive impairment, and our interview guides allowed participants to share valuable information about their experiences and perspectives. In addition to establishing feasibility for the future trial, the substantive findings identified through the qualitative interviews provide an initial understanding of the contextual factors for meal delivery and the potential mechanisms of impact across meal delivery types that warrant further examination in a full-scale trial.

## Supplementary Information

**Additional file 1.** Participant Interview Guide, Daily Meal Participants.

**Additional file 2.** Participant Interview Guide, Frozen Meal Participants.

**Additional file 3.** Caregiver Interview Guide.

## Data Availability

The datasets used and/or analyzed during the current study are available from the corresponding author on reasonable request.

## Abbreviations

PLWD: People living with dementia
TICS-M: Telephone Interview for Cognitive Status—modified
