# Supplementary material for: Feasibility of conducting qualitative research with persons living with dementia and their caregivers during a home-delivered meals pilot trial
Source: Pilot Feasibility Stud. 2023 Apr 22;9:65. doi: 10.1186/s40814-023-01302-5 (PMC10122359; doi:10.1186/s40814-023-01302-5)
Supplement: Supplementary file 2 — Additional file 2. Participant Interview Guide, Frozen Meal Participants. [file 40814_2023_1302_MOESM2_ESM.docx]

**Additional File 2**

**Participant Interview Guide, Frozen Meal Participants**

**1. Please tell me a little bit about the meals that you get at your home.**

a. If they do not mention home-delivered meals: **How do you get your meals?** (This response will cue the interviewer on how to approach the interview)

**2. What is it like to get meals delivered to your home?**

**a. Tell me a little about it: What usually happens? How does the meal get inside your home?**

*Frozen Meals - Probes about scheduling*

iv. Does someone call you to make sure you will be home when the meal(s) are

delivered?

a) When they bring your meals, does someone help you carry the box

inside?

b) Or do you carry the box yourself?

**b. What do you think about the person who brings the meals?**

*Frozen Meals - Probes about delivery person*

i. Do you get to talk to the person who brings your meals?

Is it usually the same person? (Yes, No)

1. If yes - Have you gotten to know that person? If yes - are they helpful? Are they nice to you?
2. If no - are they usually helpful?

ii. When do they come? Are they usually on time? (Yes, No)

**c. How much of the meal do you usually eat?**

*Probes about how much of the meal(s) they eat*

*i*. Do you usually eat all of each meal? Most? Only a little?

ii. Do you usually eat your meal right away? Later that day? On another day?

**d. What do you like about the meals you receive?**

*Probes about whether they like the meals or not*

i. Do you like how the meals smell? Taste? Look? (add salt; too much salt - i add it; how can you eat some of the salt).

ii. Do you get enough different types of meals?

**e. Remember, there are no right or wrong answers. What you tell us will help us know how people feel about the meals. So now tell me about what you don’t like so much about these meals.**

**f. How could they be better?**

*Probes about what they would change or keep the same*

i. Are there some meals you really like? Any you don’t like?

**3. When you don’t get meals, how do you get your food? Tell me about that.**

*Probes about where they get other meals*

i. Where do you get your meals from on weekends? (As needed: Do you go to the grocery store? Does a family member or friend bring your food? Do you get them some other way, like Mom’s Meals, grocery delivery, or takeout?)

ii. What about other meal times, like breakfast: where do you get meals from (As needed: Do you go to the grocery store? Does a family member or friend bring your food? Do you get them some other way, like Mom’s Meals, grocery delivery, or takeout?)

**4. What was it like before meals were delivered to your home?**

**a. How are these meals different from what you used to eat? Are they mostly the same as what you ate before?**

*Probes about how things are different*

i. Do you eat different types of food/meals now?

ii. Do the meals taste better, worse, or about the same as the what you used to

eat?

**b. What about your health? How do you think it is since receiving meals? Do you think your health has changed, like better, worse, or the same?**

*Probe about health*

i. Do you think your health has gotten better, worse, or about the same as

before?

**c. What about your mood? Do you think it is different since getting these meals or about the same?**

*Probe about mood*

i. Do you think your mood has gotten better, worse, or about the same as

before?

**d. In general, how is it to get meals delivered to your home?**

*Probe about how they feel*

i. Do you like getting meals delivered to your home?

**5. Do you think a friend or relative would like to get these meals too, if they could?**

**a. Why do you think so?**

*Probes about if they would recommend meals*

i. Do you like the

a) taste of the meals?

b) convenience of the meals?

ii. Does getting these meals make it easier to:

a) get enough food for the day?

b) eat healthier?

iii. Do you like

a) seeing the delivery person?

b) talking to the delivery person?

**b. What about things that are not so good?**

*Probes about what they don’t like about meals*

i. Do you not like the

a) taste of the meals?

b) how often the meals are delivered?

c) anything else?

**c. What else do you want to tell us about getting meals delivered to your home?**
