# Supplementary material for: Feasibility of conducting qualitative research with persons living with dementia and their caregivers during a home-delivered meals pilot trial
Source: Pilot Feasibility Stud. 2023 Apr 22;9:65. doi: 10.1186/s40814-023-01302-5 (PMC10122359; doi:10.1186/s40814-023-01302-5)
Supplement: Supplementary file 3 — Additional file 3. Caregiver Interview Guide. [file 40814_2023_1302_MOESM3_ESM.docx]

**Additional File 3**

**Caregiver Interview Guide**

1. **Tell me a bit more about yourself. What is your relationship to [meal recipient’s name]?**

*Probes about participant*

i. Do you live with [meal recipient’s name]?

ii. How long have you been helping them with day-to-day tasks?

iii. What sort of things do you help [meal recipient’s name] with?

iv. How often do you help [meal recipient’s name]? every day? number of hours?

1. **Tell me what it’s like for [meal recipient’s name] to get meals delivered.**

*Probes about meal delivery*

i. Are you usually there when the meals are delivered?

ii. How does [meal recipient’s name] act with the person who brings the meals?

iii. How does the [meal recipient’s name] prepare the meals?

iv. If someone helps, who is it and how do they help?

v. How much of the meal is eaten?

vi. What does [meal recipient’s name] like/not like about receiving the meals?

vii. When [meal recipient’s name] does not receive meals, how do they get their food?

For example, on weekends? Or other times?

1. **Think about before meals were coming to [meal recipient’s name] home and then what it was like after they started to get meals.**

*Probes about time before receiving meals*

i. How do you think the meals compare to what [meal recipient’s name] used to eat?

ii. How do you think the meals have affected [meal recipient’s name] health?

iii. How has getting these meals at home affected [meal recipient’s name] ability to continue to live at home?

1. **We’ve heard about how meals affected [meal recipient’s name]. What about how meal delivery for [meal recipient’s name] has affected YOUR life.**

*Probes about how meal delivery has impacted their life*

i. Are there ways that the meal delivery has made your life more positive or easier?

ii. Are there ways that the meal delivery has made your life more challenging or stressful?

iii. Has it affected your health or mood? feelings of isolation? If so, how?

iv. Has it affected how you think about safety or finances? If so, how?

v. Has it affected your ability to continue providing care? If so, how?

1. **We hope you can suggest how the meal program could be improved especially for people who have dementia, memory loss or cognitive impairment?**

*Probes about changes to meal program*

i. What about other ways to communicate with [your meal recipient]? meal recipients more generally? caregivers about clients?

ii. What about other types of assistance or resources that could be provided to clients with dementia, memory loss, or cognitive impairment? If so, what would help?

1. **We would like to get demographic information from caregivers to help us better understand any possible differences in people’s experiences. Would you be willing to share your:**

- age,
- gender,
- race and ethnicity, and
- whether or not you are currently working full-time or part-time?

1. **Tell me a bit more about yourself. What is your relationship to [meal recipient’s name]?**

*Probes about participant*

i. Do you live with [meal recipient’s name]?

ii. How long have you been helping them with day-to-day tasks?

iii. What sort of things do you help [meal recipient’s name] with?

iv. How often do you help [meal recipient’s name]? every day? number of hours?

1. **Tell me what it’s like for [meal recipient’s name] to get meals delivered.**

*Probes about meal delivery*

i. Are you usually there when the meals are delivered?

ii. How does [meal recipient’s name] act with the person who brings the meals?

iii. How does the [meal recipient’s name] prepare the meals?

iv. If someone helps, who is it and how do they help?

v. How much of the meal is eaten?

vi. What does [meal recipient’s name] like/not like about receiving the meals?

vii. When [meal recipient’s name] does not receive meals, how do they get their food?

For example, on weekends? Or other times?

1. **Think about before meals were coming to [meal recipient’s name] home and then what it was like after they started to get meals.**

*Probes about time before receiving meals*

i. How do you think the meals compare to what [meal recipient’s name] used to eat?

ii. How do you think the meals have affected [meal recipient’s name] health?

iii. How has getting these meals at home affected [meal recipient’s name] ability to continue to live at home?

1. **We’ve heard about how meals affected [meal recipient’s name]. What about how meal delivery for [meal recipient’s name] has affected YOUR life.**

*Probes about how meal delivery has impacted their life*

i. Are there ways that the meal delivery has made your life more positive or easier?

ii. Are there ways that the meal delivery has made your life more challenging or stressful?

iii. Has it affected your health or mood? feelings of isolation? If so, how?

iv. Has it affected how you think about safety or finances? If so, how?

v. Has it affected your ability to continue providing care? If so, how?

1. **We hope you can suggest how the meal program could be improved especially for people who have dementia, memory loss or cognitive impairment?**

*Probes about changes to meal program*

i. What about other ways to communicate with [your meal recipient]? meal recipients more generally? caregivers about clients?

ii. What about other types of assistance or resources that could be provided to clients with dementia, memory loss, or cognitive impairment? If so, what would help?

1. **We would like to get demographic information from caregivers to help us better understand any possible differences in people’s experiences. Would you be willing to share your:**

- age,
- gender,
- race and ethnicity, and
- whether or not you are currently working full-time or part-time?
